# Prediction of all-cause mortality after liver transplantation using left ventricular systolic and diastolic function assessment

**DOI:** 10.1371/journal.pone.0209100

**Published:** 2019-01-25

**Authors:** Young-Jin Moon, Jung-Won Kim, Yun-Sic Bang, Young Su Lim, Yumin Ki, Bo-Hyun Sang

**Affiliations:** 1 Department of Anesthesiology and Pain Medicine, Laboratory for Cardiovascular Dynamics, Asan Medical Center, University of Ulsan College of Medicine, Seoul, Republic of Korea; 2 Department of Anesthesiology and Pain Medicine, Catholic Kwandong University of Korea College of Medicine, International St. Mary's Hospital, Incheon, Republic of Korea; 3 Department of Anesthesiology and Pain Medicine, CHA Bundang Medical Center, CHA University, Seongnam, Republic of Korea; Ospedale del Cuore G Pasquinucci Fondazione Toscana Gabriele Monasterio di Massa, ITALY

## Abstract

Although pretransplant cardiac dysfunction is considered a major predictor of poor outcomes after liver transplantation (LT), the ability of left ventricular (LV) systolic/diastolic function (LVSF/LVDF), together or individually, to predict mortality after LT is poorly characterized. We retrospectively evaluated pretransplant clinical and Doppler echocardiographic data of 839 consecutive LT recipients from 2009 to 2012 aged 18–60 years. The primary endpoint was all-cause mortality at 4 years. The overall survival rate was 91.2%. In multivariate Cox analysis, reduced LV ejection fraction (LVEF, *P* = 0.014) and decreased transmitral E/A ratio(*P* = 0.022) remained significant prognosticators. In LVSF analysis, patients with LVEF≤60% (quartile [Q]1) had higher mortality than those with LVEF>60% (hazard ratio = 1.90, 95% confidence interval = 1.15–3.15, *P* = 0.012). In LVDF analysis, patients with an E/A ratio<0.9(Q1) had a 2.19-fold higher risk of death (95% confidence interval = 1.11–4.32, *P* = 0.024) than those with an E/A ratio>1.4(Q4). In combined LVDF and LVSF analysis, patients with an E/A ratio<0.9 and LVEF≤60% had poorer survival outcomes than patients with an E/A ratio≥0.9 and LVEF>60% (79.5% versus 93.3%, *P* = 0.001). Patients with an early mitral inflow velocity/annular velocity (E/e’ ratio)>11.5(Q4) and LV stroke volume index (LVSVI)<33mL/m^2^(Q1) showed worse survival than those with an E/e’ ratio≤11.5 and LVSVI ≥33mL/m^2^(78.4% versus 92.2%, *P* = 0.003). A combination of LVSF and LVDF is a better predictor of survival than LVSF or LVDF alone.

## Introduction

Recent advances in surgical techniques and better immunosuppressive drug therapy-related perioperative management have improved graft-related survival after liver transplantation (LT). Thus, in the current LT era, pretransplantation cardiovascular disease is emphasized as the leading cause of poor nongraft-related short- and long-term outcomes [1–3].

Doppler echocardiography is highly recommended before LT and is now routine practice at many institutions for the assessment of left and right ventricular dysfunction, pulmonary hypertension, and cirrhotic cardiomyopathy [4]. Patients with the latter condition primarily show left ventricular diastolic dysfunction (LVDD) with normal systolic function at rest but an impaired contractile response to stress [5, 6]. LVDD is currently acknowledged as one of the major causes of peritransplant heart failure and of poorer early and late outcomes in patients who undergo LT [1, 7–9].

However, the aforementioned results have mostly been based on limited diastolic/systolic parameters or an incomplete diastolic grading system that appears to be somewhat discordant [7–9]. This is likely to be because only a few studies have examined the echocardiographic characteristics of LT candidates in a large cohort. In addition, there is no clear classification of the cardiac dysfunction of end-stage liver disease (ESLD) patients, who typically show hyperdynamic circulation with high cardiac output. The E/e’ ratio, an index if LV filling pressure, is one of the main echocardiographic parameters of LVDD. Interestingly, previous studies revealed that an E/e’ ratio>10 is highly associated with pretransplant mortality at 1 year and early development of heart failure after LT [7, 10]. However, this cut-off value is not high enough, when compared to non-ESLD patients of E/e’ ratio ≥15 [11], suggesting that there may be considerable differences in the normal values of echocardiographic parameters between ESLD and non-ESLD patients.

No studies have systematically evaluated whether the quantitative echocardiographic parameters can predict mortality in LT candidates. Accordingly, using a sufficiently-sized study cohort, we aimed to examine whether the quantitative echocardiographic parameters are applicable to the outcome prediction and stratify the cardiac risk of LT candidates using pretransplant Doppler echocardiography in order to predict all-cause mortality after LT. The Doppler echocardiography parameters quantitatively analyzed were left ventricular systolic function (LVSF) alone, left ventricular diastolic function (LVDF) alone, or LVSF and LVDF together.

## Methods

### Patients and follow-up

We retrospectively evaluated prospectively collected data of 964 consecutive patients aged 18–60 years who underwent LT at Asan Medical Center, Seoul, Republic of Korea, between October 2009 and October 2012. We excluded LT candidates older than 60 years of age because the frequency of LVDD in this population is high [10, 12]. Preoperative Doppler echocardiography was routinely performed in the Asan Medical Center echocardiography laboratory. Of the 964 patients, 125 were excluded from the analysis: 80 who had an incomplete or absent echocardiographic study, 24 who underwent re-transplantation, 13 who had coexisting chronic renal failure (glomerular filtration rate <60 mL∙min^-1^∙1.73m^-2^ by Modified Diet in Renal Disease equation or need for hemodialysis), and 8 who had history of open heart surgery or significant valvular heart disease (greater than a moderate degree of valvular stenosis/regurgitation). Thus, 839 recipients of primary LT were included in our final study series. Demographic, laboratory, and clinical data from routine preoperative evaluations were collected by analysis of electronic medical records after approval of the Institutional Review Board of Asan Medical Center. The primary endpoint was 4-year all-cause mortality. All patients were followed for at least 1 year and up to 4 years. Mortality data were collected from the electronic medical records and our hospital’s registry, which is updated by the Asan Organ Transplantation Center.

### Echocardiographic measurements

Prior to surgery, preoperative echocardiography at our institution’s echocardiography laboratory was performed routinely by experienced and well-trained sonographers and was reconfirmed by 3 attending staff cardiologists. Comprehensive two-dimensional and Doppler echocardiography with tissue Doppler imaging (TDI) were performed using a Hewlett-Packard Sonos 2500 or 5500 imaging system equipped with a 2.5-MHz transducer (Hewlett-Packard, Andover, MA) to evaluate cardiac morphology and function. Parameters measured included end-systolic LV posterior wall thickness, end-diastolic LV posterior wall thickness, end-systolic interventricular septum thickness, end-diastolic interventricular septum thickness, left ventricular mass index, left atrial diameter, and aortic diameter. LV end-diastolic volume (EDV) and end-systolic volume (ESV) were measured using the Teichholz method or biplane modified Simpson’s rule, as appropriate, from which stroke volume (SV = EDV–ESV) and LV ejection fraction (LVEF) were calculated. Measurements were indexed to body surface area when appropriate. We measured the peak transmitral inflow velocity (E and A) on pulsed-wave Doppler, E wave deceleration time (DT) to assess restriction to LV filling, and the E/A ratio from the apical four-chamber view. The systolic s’ and diastolic e’ and a’ peak velocities were obtained by TDI at the septal mitral annulus on the apical four-chamber view, and the e’/a’ and E/e’ ratios as an estimation of LV filling pressure were calculated according to established guidelines [12, 13]. The peak systolic tricuspid regurgitation gradient was also measured.

Global LV systolic function was assessed using (1) TDI s’ velocity, (2) ejection fraction (EF), (3) fractional shortening, and (4) LV SV index (LVSVI). Diastolic function was assessed using (1) the E/A ratio, (2) DT, (3) TDI septal e’ velocity, (4) E/e’ ratio, and (5) e’/a’ ratio. Combined diastolic and systolic function was assessed by (1) echocardiographic parameters showing *P*<0.1 in univariate analysis for predicting mortality and (2) multiple combinations of systolic and diastolic parameters that are known to be clinically relevant [10, 14], such as (1) the E/e′ ratio and LVSVI and (2) the E/A ratio and LVEF, as appropriate.

### Statistical analysis

Normally distributed continuous data were expressed as the mean ± standard deviation or, if skewed, as median values and interquartile range. Categorical data were expressed as numbers and percentages. LT recipients were divided into two groups, survivors and non-survivors at the end of a 4-year follow-up. According to the normality assumptions, a Student *t* test, nonparametric Wilcoxon signed-rank test, chi-square test, or Fisher exact test was used for comparisons of continuous or categorical variables, as appropriate. Univariate Cox proportional hazards analysis was performed to identify variables associated with all-cause mortality. The variables with *P*<0.1 in univariate Cox analysis were included in the multivariate Cox proportional hazards analysis. Age and sex were forced into the final multivariable model because they are known risk factors for mortality. Furthermore, the influence of age on Doppler echocardiographic indices of LVDF, such as the E/A ratio, is well recognized [15].

Multivariate Cox proportional hazards stepwise inclusion/exclusion regression modeling with backward elimination (probability for removal of 0.10) was used to identify independent prognostic variables and to calculate adjusted hazard ratios with 95% confidence intervals (CIs). The proportional hazards assumption was confirmed by examination of log (-log [survival]) curves. Additionally, to stratify the cardiac risk of LT candidates with quantitative analysis and to compare survival among groups, final echocardiographic variables remaining in the multivariable model were categorized into di-, tri-, or quadrichotomized distributions, and then the statistical significance of arbitrary cut-off points derived from the median, tertile, and quartile analyses was combined and rigorously validated. Also, to exclude significant effects of multicollinearity among measures of systolic function, diastolic function, and combined systolic and diastolic function, separate models were constructed. Namely, statistically significant clinical variables in univariate analysis (*P*<0.05) were equally added to adjust the systolic function (model 1), diastolic function (model 2), and combined systolic and diastolic function (models 3 and 4), respectively. By using these models, hazard ratios with 95% CI for all-cause mortality were determined and compared. Survival analysis to compare the mortality among subgroups was performed using the Kaplan-Meier method with log-rank statistic. All statistical analyses were performed using SPSS version 21.0 software (SPSS Inc., Chicago, IL). Differences were considered significant if *P* was less than 0.05.

## Results

### Clinical characteristics and laboratory data

The preoperative clinical characteristics and laboratory data of the 839 patients are listed in Table 1. The hemodynamic and echocardiographic variables are summarized in Table 2. Table 3 presents the clinical and echocardiographic findings of those who survived and did not survive. Among all patients, 74 patients (8.8%) had died by the end of the 4-year follow-up (28.3±12.1 months). The most two common cause of death were septic shock (27.0%) and recurrence of hepatocellular carcinoma (21.6%) followed by primary non-function of graft (14.9%) and multiorgan failure (5.4%). The overall survival rate was 97.1% at 3 months, 93.6% at 1 year, and 91.2% at 4 years after LT.

**Table 1:** Clinical characteristics of the study patients.

Variables		n = 839
Age (years)		51 (46–55)
Male		644 (76.8%)
Body mass index (kg/m^2^)		23.8 (21.6–26.3)
Hypertension		97 (11.6%)
Diabetes mellitus		177 (21.1%)
Ischemic heart disease		112 (13.3%)
Varix bleeding history		199 (23.7%)
MELD score		14 (10–23)
Child-Turcotte-Pugh score		8 (6–10)
Child-Turcotte-Pugh class (A/B/C)		237/291/311 (28.2%/34.7%/37.1%)
Medication		
	Diuretics	234 (27.9%)
	Beta blockers	144 (17.2%)
Cause of liver disease		
	Virus-related cirrhosis	622 (74.1%)
	Hepatitis B/C virus	579 (69.0%)/43 (5.1%)
	Alcoholic cirrhosis	108 (12.9%)
	Toxic hepatitis	35 (4.2%)
	Others	74 (8.8%)
Donor type		
	Cadaveric	112 (13.3%)
	Living	727 (86.7%)
	Right/Left liver graft	667 (91.7%)/7 (1.0%)
	Dual donor	53 (7.3%)
	Graft-recipient weight ratio	0.98 (0.84–1.16)
Laboratory data		
	Hemoglobin (g/dL)	10.6 (9.1–12.4)
	Platelet (×10^3^/μl)	58 (39–84)
	Total bilirubin (mg/dL)	2.6 (1.3–9.4)
	Albumin (g/dL)	3.1 (2.7–3.6)
	Creatinine (mg/dL)	0.8 (0.6–1.0)
	Prothrombin time (INR)	1.45 (1.21–1.89)
	BNP (pg/mL)	46 (19–111)
	Log BNP (pg/mL)	3.9 ± 1.3

Data are expressed as median (interquartile range), mean ± standard deviation, or number (percentage).

Abbreviations: MELD, Model for End-stage Liver Disease; INR, international normalized ratio; BNP, B-type natriuretic peptide.

**Table 2:** Hemodynamic and echocardiographic data.

Variables		n = 839
Hemodynamic data		
	Systolic blood pressure (mmHg)	110 (100–121)
	Diastolic blood pressure (mmHg)	70 (62–77)
	QTc (ms)	449 (429–470)
	QTc over 450 ms in males	83/195 (42.6%)
	QTc over 460 ms in females	291/644 (45.2%)
Echocardiographic data		
	LVEDV index (mL/m^2^)	61.5 (52.5–71.8)
	LVESV index (mL/m^2^)	21.8 (18.1–26.1)
	LVMI (g/m^2^)	89.7 (78.3–102.9)
	PGsys (RV-RA) (mmHg) (n = 711)	21.0 (19.0–27.0)
Systolic function		
	Fractional shortening (%)	41.0 ± 6.1
	LVSV index (mL/m^2^)	39.2 (33.2–45.6)
	LVEF (%)	64.0 (61.0–67.0)
	≤60	160 (19.1%)
	<55	2 (0.2%)
	s’ (cm/s)	8.4 (7.5–9.7)
Diastolic function		
	LA diameter index (mm/m^2^)	22.5 (20.6–24.7)
	E/A ratio	1.14 (0.92–1.44)
	DT (ms)	201 (177–230)
	e’ (cm/s)	7.8 (6.5–9.1)
	a’ (cm/s)	9.0 (7.9–10.8)
	e’/a’ ratio	0.81 (0.66–1.08)
	E/e’ ratio	9.5 (8.0–11.5)

Data are expressed as median (interquartile range), mean ± standard deviation, or number (percentage).

Abbreviations: QTc, corrected QT interval; LVMI, left ventricular mass index; PGsys (RV-RA), systolic pressure gradient between right ventricle and right atrium; LVEDV, left ventricular end-diastolic volume; LVESV, left ventricular end-systolic volume; LVSV, left ventricular stroke volume; LVEF, left ventricular ejection fraction; s’, systolic myocardial velocity; LA, left atrium; E, early transmitral flow velocity; A, late transmitral flow velocity; DT, deceleration time of E; e’, early diastolic myocardial velocity; a’, late diastolic myocardial velocity.

**Table 3:** Univariate cox regression analysis of survivors and non-survivors.

Variables		Survivors (n = 765)	Non-survivors (n = 74)	Hazard ratio (95% CI)	*P*
Age (years)		51 (47–55)	52 (47–56)	1.02 (0.99–1.05)	0.240
Male		588 (76.9%)	56 (75.7%)	1.05 (0.62–1.78)	0.860
Body mass index (kg/m^2^)		23.8 (21.7–26.2)	23.3 (20.8–26.4)	0.98 (0.92–1.05)	0.613
Hypertension		86 (11.2%)	11 (14.9%)	0.77 (0.40–1.45)	0.415
Diabetes mellitus		162 (21.2%)	15 (20.3%)	1.08 (0.61–1.90)	0.802
Ischemic heart disease		105 (13.7%)	7 (9.5%)	1.44 (0.66–3.13)	0.360
Varix bleeding history		183 (23.9%)	16 (21.6%)	1.13 (0.65–1.97)	0.655
MELD score		14 (9–21)	19 (12–29)	1.03 (1.01–1.05)	0.001
Child-Turcotte-Pugh score		8 (6–10)	9 (7–12)	1.12 (1.03–1.22)	0.010
Child-Turcotte- Pugh class					
	A	223 (29.2%)	14 (18.9%)	1.00	
	B	264 (34.5%)	27 (36.5%)	1.58 (0.83–3.01)	0.167
	C	278 (36.3%)	33 (44.6%)	1.82 (0.98–3.41)	0.060
Medication					
	Diuretics	214 (28.0%)	20 (27.0%)	1.06 (0.63–1.77)	0.833
	Beta blockers	135 (17.6%)	9 (12.2%)	1.54 (0.77–3.08)	0.228
Donor type					
	Cadaveric	97 (12.7%)	15 (20.3%)	1.71 (0.97–3.01)	0.065
	Living	668 (87.3%)	59 (79.8%)	1.00	
	Graft-recipient weight ratio	0.98 (0.85–1.115)	1.04 (0.89–1.19)	2.01 (0.76–5.36)	0.162
Hemodynamic data					
	Systolic blood pressure (mmHg)	110 (100–121)	111 (101–121)	1.00 (0.99–1.02)	0.597
	Diastolic blood pressure (mmHg)	70 (62–77)	68 (63–75)	1.00 (0.98–1.02)	0.996
	QTc (ms)	448 (428–469)	448 (430–470)	1.00 (0.99–1.01)	0.964
Laboratory data					
	Hemoglobin (g/dL)	10.7 (9.2–12.4)	10.0 (8.7–11.8)	0.94 (0.85–1.05)	0.267
	Platelet (×10^3^/μl)	57 (38–84)	58 (38.5–89.5)	1.00 (1.00–1.01)	0.290
	Total bilirubin (mg/dL)	2.6 (1.3–7.6)	4.4 (1.7–20.7)	1.02 (1.00–1.04)	0.023
	Albumin (g/dL)	3.1 (2.7–3.6)	3.2 (2.9–3.7)	1.11 (0.80–1.54)	0.523
	Creatinine (mg/dL)	0.80 (0.60–0.98)	0.90 (0.70–1.61)	1.34 (1.16–1.54)	<0.001
	Prothrombin time (INR)	1.45 (1.21–1.85)	1.51 (1.21–2.01)	1.20 (0.91–1.57)	0.193
	BNP (pg/mL)	45 (19–109)	61 (20–158)	1.00 (1.00–1.00)	0.115
	Log BNP (pg/mL)	3.9 ± 1.3	4.1 ± 1.5	1.12 (0.94–1.32)	0.196
Echocardiographic data					
	LVEDV index (mL/m^2^)	61.6 (52.7–72.0)	60.1 (52.3–70.4)	1.00 (0.98–1.01)	0.497
	LVESV index (mL/m^2^)	21.9 (18.3–26.2)	21.7 (18.3–26.1)	1.01 (0.97–1.05)	0.607
	LVMI (g/m^2^)	90.3 (78.4–102.8)	90.4 (78.5–105.0)	1.00 (0.99–1.02)	0.474
	PGsys (RV-RA) (mmHg) (n = 711)	21.0 (19.0–27.0)	23.0 (18.0–27.0)	1.02 (0.98–1.06)	0.463
Systolic function					
	Fractional shortening (%)	41.0 ± 6.1	41.0 ± 5.7	1.00 (0.96–1.04)	0.960
	LVSV index (mL/m^2^)	39.4 (33.4–46.1)	38.4 (31.8–44.2)	0.98 (0.96–1.01)	0.102
	LVEF (%)	65.0 (61.0–67.0)	63.0 (60.0–66.0)	0.94 (0.89–0.99)	0.025
	s’ (cm/s)	8.4 (7.4–9.6)	8.2 (7.2–9.7)	1.07 (0.95–1.20)	0.258
Diastolic function					
	LA diameter index (mm/m^2^)	22.6 (20.6–24.7)	22.5 (20.9–25.1)	0.96 (0.90–1.03)	0.276
	E/A ratio	1.2 (0.9–1.5)	1.0 (0.9–1.3)	0.43 (0.22–0.83)	0.012
	DT (ms)	201 (179–230)	210 (173–235)	1.00 (1.00–1.01)	0.175
	e’ (cm/s)	7.8 (6.6–9.1)	7.1 (6.0–9.1)	0.88 (0.77–1.00)	0.045
	a’ (cm/s)	9.0 (7.9–10.6)	8.8 (7.9–10.9)	1.04 (0.94–1.15)	0.469
	e’/a’ ratio	0.8 (0.7–1.1)	0.8 (0.6–1.0)	0.49 (0.22–1.09)	0.079
	E/e’ ratio	9.5 (8.0–11.5)	9.9 (8.7–12.0)	1.08 (1.00–1.15)	0.043

Data are expressed as median (interquartile range), mean ± standard deviation or number (percentage).

Abbreviations: MELD, Model for End-stage Liver Disease; QTc, corrected QT interval; INR, international normalized ratio; BNP, brain natriuretic peptide; LVMI, left ventricular mass index; PGsys (RV-RA), systolic pressure gradient between right ventricle and right atrium; LVEDV, left ventricular end-diastolic volume; LVESV, left ventricular end-systolic volume; LVSV, left ventricular stroke volume; LVEF, left ventricular ejection fraction; s’, systolic myocardial velocity; LA, left atrium; E, early transmitral flow velocity; A, late transmitral flow velocity; DT, deceleration time of E; e’, early diastolic myocardial velocity; a’, late diastolic myocardial velocity.

### Echocardiographic characteristics of ESLD patients

**Fig 1:**
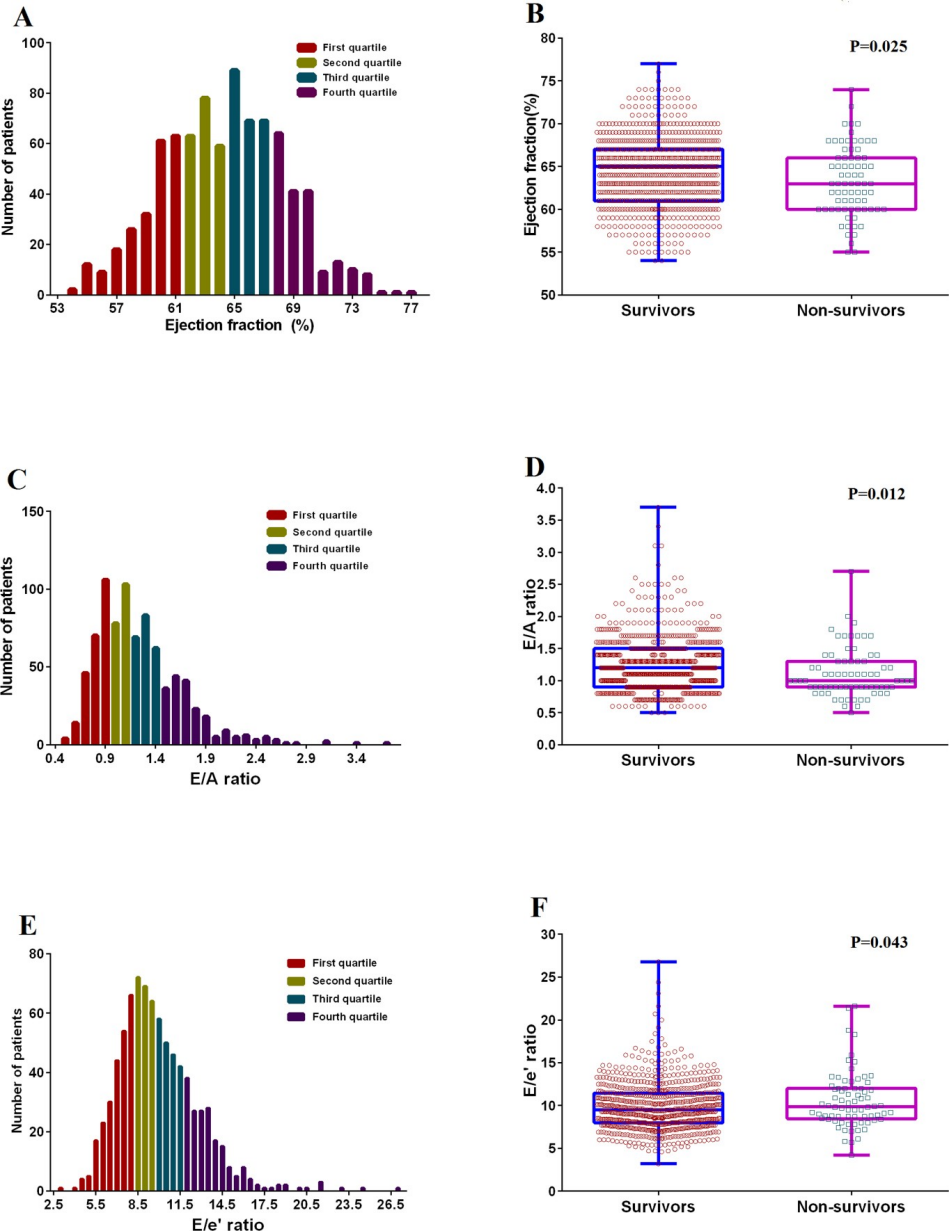
Histograms show patients divided into quartiles according to (A) LVEF, (C) E/A ratio, and (E) E/e’ ratio. Box plots depict (B) LVEF, (D) E/A ratio, and (F) E/e’ ratio in survivors and non-survivors. The bottom and top edges of the boxes are the 25^th^ and 75^th^ percentiles, respectively. Median values are shown by the line whthin the box. All values are shown individually (open circles and squares). LVEF, left ventricular ejection fraction.

In the analysis of systolic function among all patients, the cut-off values of the lowest quartile (Q1, 25^th^ percentile) and 10^th^ and 5^th^ percentiles of LVEF were 61%, 59%, and 58%, respectively (Fig 1). 1.7% of all patients had LVEF≤55% and only 0.2% had LVEF<55%. All patients had LVEF>50%. The Q1 values of LVSVI and TDI s' were 33.2mL/m^2^ and 7.5cm/s, respectively, and their 5^th^ percentiles were 25.9mL/m^2^ and 6.3cm/s, respectively.

In quartile analysis of diastolic parameters, the Q1 cut-off values of the E/A ratio and DT were 0.9 and 177 ms, respectively. The cut-off value of the highest quartile (Q4) of the E/e' ratio was 11.5 in this study cohort (Fig 1).

### Predictors of overall late mortality

Among all clinical and laboratory variables in univariate Cox analysis, only the Child-Turcotte-Pugh (CTP) score (*P* = 0.01), Model for End-stage Liver Disease (MELD) score (*P* = 0.001), serum creatinine (*P*<0.001), and total bilirubin level (*P* = 0.023) significantly predicted 4-year mortality (Table 3). To avoid significant effects of multicollinearity between CTP and MELD scores, only the CTP score was entered into the multivariable analysis. In systolic function analysis using univariate Cox analysis, there was a significant decrease in LVEF in non-survivors (median, 65.0% versus 63.0%, *P* = 0.025), but not in TDI s’ velocity, fractional shortening, or LVSVI. In diastolic function analysis, non-survivors showed a decreased E/A ratio (median, 1.0 versus 1.2, *P* = 0.012), reduced e’ velocity (median, 7.1cm/s versus 7.8cm/s, *P* = 0.045), and increased E/e’ ratio (median, 9.9 versus 9.5, *P* = 0.043) compared with survivors at 4 years (Table 3, Fig 1).

When all clinical and echocardiographic variables showing *P*<0.1 (i.e., CTP score, donor type, total bilirubin, creatinine, LVEF, E/A ratio, septal e’, e’/a’ ratio, and E/e’ ratio) were entered into the Cox multivariate survival analysis, an increased serum creatinine level and CTP score (Wald statistic = 8.4 and 4.5, *P* = 0.004 and *P* = 0.034, respectively) and reduced EF and E/A ratio (Wald statistic = 6.1 and 5.2, *P* = 0.014 and *P* = 0.022, respectively) were the only predictors of 4-year all-cause mortality after LT (Table 4). The other clinical variables did not show statistical significance.

**Table 4:** Univariate and multivariate cox regression analysis of predictors of 4-year survival.

	Univariate Analysis		Multivariate Analysis*	
Variables	Hazard ratio (95%CI)	*P* Value	Hazard ratio (95%CI)	*P* Value
Child-Turcotte-Pugh score	1.12 (1.03–1.22)	0.010	1.10 (1.01–1.20)	0.034
Donor type (cadaver)	1.71 (0.97–3.01)	0.065		
Total bilirubin (mg/dL)	1.02 (1.00–1.04)	0.023		
Creatinine (mg/dL)	1.34 (1.16–1.54)	<0.001	1.25 (1.08–1.45)	0.004
LVEF (%)	0.94 (0.89–0.99)	0.025	0.93 (0.88–0.99)	0.014
E/A ratio	0.43 (0.22–0.83)	0.012	0.47 (0.24–0.90)	0.022
e’ (cm/s)	0.88 (0.77–1.00)	0.045		
e’/a’ ratio	0.49 (0.22–1.09)	0.079		
E/e’ ratio	1.08 (1.00–1.15)	0.043		

*Adjusted for age and sex. Abbreviations: LVEF, left ventricular ejection fraction; E, early transmitral flow velocity; A, late transmitral flow velocity; e’, early diastolic myocardial velocity; a’, late diastolic myocardial velocity.

### Systolic, diastolic, and combined cardiac function and late mortality according to quartile analysis

**Fig 2:**
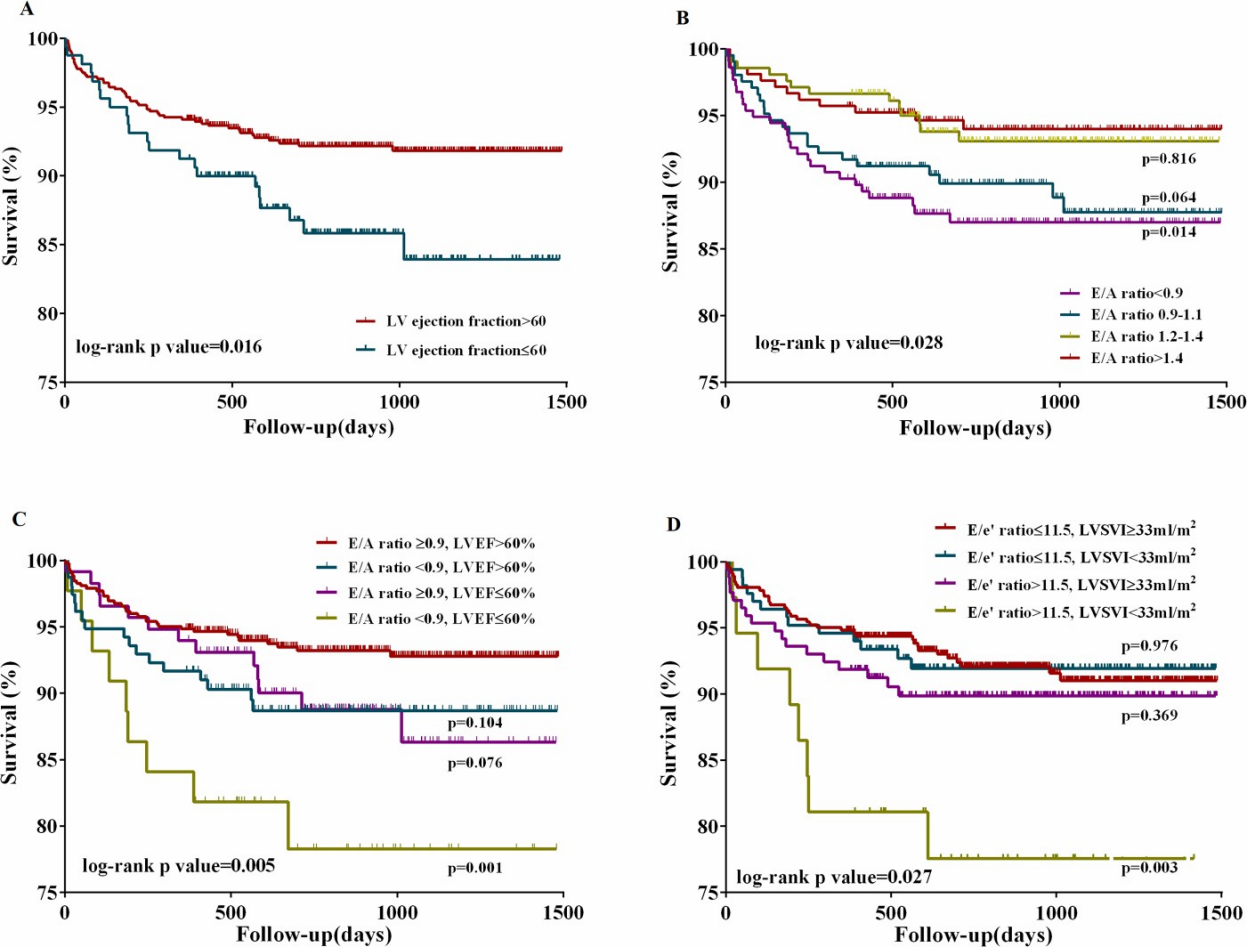
Kaplan-Meier survival analysis assessed by quantitative Doppler echocardiography. (A) The survival rate of patients with LVEF ≤60% was significantly lower than that of those with LVEF >60% (log-rank test, *P* = 0.016). (B) The survival rates of patients were significantly different among groups according to the E/A ratio quartile (log-rank test, *P* = 0.028). (C) The survival rates of patients were significantly different among groups according to the combined criteria (the E/A ratio quartile and LVEF) (log-rank test, *P* = 0.005). (D) The survival rates of patients were significantly different among groups according to the combined criteria (the E/e’ ratio quartile and LVSVI) (log-rank test, *P* = 0.027). LVEF, left ventricular ejection fraction; LVSVI, left ventricular stroke volume index.

Because only a reduced EF and decreased E/A ratio were independent predictors of 4-year mortality, they were dichotomized and quadrichotomized to stratify cardiac risks according to cut-off values derived from the quartile analysis of this study cohort, as appropriate. When dichotomizing the patients by LVEF≤60% (Q1), the 4-year mortality rate was significantly lower in the LVEF>60% group than in the LVEF≤60% group (7.7% and 13.8%, respectively; *P* = 0.015). Kaplan-Meier survival curve showed this difference between two groups (log-rank test, *P* = 0.016, Fig 2A). However, no differences between groups were seen at 3 months (2.8% and 3.1%, respectively; *P* = 0.824) and 1 year (5.9% and 8.8%, respectively; *P* = 0.185) (S1 Table).

In subgroup analysis with E/A ratio quartiles (S1 Table), 4-year survival rates were 87.5%, 89.3%, 93.7%, and 94.3% in Q1, Q2, Q3 and Q4, respectively (*P* = 0.004). Kaplan-Meier survival curve showed this difference among groups according to the E/A ratio quartile (log-rank test, *P* = 0.028, Fig 2B). The clinical and echocardiographic characteristics of all patients according to their E/A ratio quartiles and by LVEF≤60% and LVEF>60% groups are summarized in S1 Table.

Additionally, we calculated adjusted hazard ratios by separating the models according to LVSF, LVDF, and combined LVSF and LVDF (Table 5). In model 1, when LVEF≤60% was adjusted with age, sex, and clinically significant variables (CTP score, total bilirubin level, creatinine level), LVEF≤60% (hazard ratio = 1.90, 95% CI = 1.15–3.15, *P* = 0.012) predicted 4-year mortality independently of the CTP score and creatinine level.

**Table 5:** Multivariate cox analysis of echocardiographic variables according to left ventricular systolic/diastolic function alone or together.

	Hazard ratio (95%CI)	Wald	*P*
**Model 1: Systolic function**			
LV ejection fraction ≤60%	1.90 (1.15–3.15)	6.3	0.012
**Model 2: Diastolic function**			
E/A ratio by quartiles		8.0	
<0.9	2.19 (1.11–4.32)		0.024
0.9–1.1	1.89 (0.94–3.82)		0.076
1.2–1.4	1.07 (0.49–2.34)		0.869
>1.4	1.00		
**Model 3: Combined function (E/A ratio with LV ejection fraction)**			
E/A ratio ≥0.9, LV ejection fraction >60%	1.00	11.0	
E/A ratio <0.9, LV ejection fraction >60%	1.59 (0.89–2.84)		0.116
E/A ratio ≥0.9, LV ejection fraction ≤60%	1.72 (0.91–3.28)		0.097
E/A ratio <0.9, LV ejection fraction ≤60%	3.26 (1.56–6.80)		0.002
**Model 4: Combined function (E/e’ ratio with LV stroke volume index)**			
E/e’ ratio ≤11.5, LV stroke volume index ≥33 mL/m^2^	1.00	8.0	
E/e’ ratio >11.5, LV stroke volume index ≥33 mL/m^2^	1.16 (0.65–2.08)		0.616
E/e’ ratio ≤11.5, LV stroke volume index <33 mL/m^2^	1.05 (0.56–1.99)		0.873
E/e’ ratio >11.5, LV stroke volume index <33 mL/m^2^	2.98 (1.38–6.43)		0.005

Model 1,2,3 and 4 are adjusted for age, sex, Child-Turcotte-Pugh score, total bilirubin and creatinine,respectively.

Abbreviations: LV, left ventricular; E’, early diastolic myocardial velocity; E, early transmitral flow velocity; A, late transmitral flow velocity.

In model 2, when quartiles of the E/A ratio were adjusted for age, sex, and clinically significant variables, E/A ratio quartiles were independent predictors of late mortality. Patients in E/A ratio Q1 (<0.9) had a 2.19-fold higher risk of death (95% CI = 1.11–4.32, *P* = 0.024) than those in E/A ratio Q4 (>1.4), independently of the creatinine level.

To identify significant parameters in combined LVDF and LVSF analysis, multiple combinations of the LVEF, LVSVI, E/A ratio, and E/e’ ratio were performed using the cut-off values of quartile analysis. Therefore, in model 3, the combined LVDF and LVSF analysis, patients were divided into four subgroups according to their E/A ratio Q1 (0.9) and LVEF Q1 (60%) cut-off values and were also adjusted for age, sex, and clinically significant variables (Table 5). Patients (*n* = 44, 5.2%) with a combined E/A ratio<0.9 and LVEF≤60% had a worse 4-year survival than patients with an E/A ratio≥0.9 and LVEF>60% (79.5% versus 93.3%, *P* = 0.001). Kaplan-Meier survival curve showed this significant difference among groups according to the combined criteria (the E/A ratio quartile and LVEF) (log-rank test, P = 0.005, Fig 2C). Additionally, although a single parameter of LVSVI did not show statistical significance in univariate analysis (*P* = 0.102), LVSVI was significant when combined with the E/e’ ratio (*P* = 0.043 in univariate analysis). Therefore, in model 4, the combined LVDF and LVSF analysis, patients were divided into four subgroups according to the LVSVI Q1 (33mL/m^2^) and E/e’ ratio Q4 (11.5) cut-off values and were also adjusted for age, sex, and clinically significant variables (Table 5). Patients (*n* = 37, 4.4%) with a combined E/e’ ratio>11.5 and SVI<33mL/m^2^ showed worse survival than those with an E/e’ ratio≤11.5 and SVI ≥33 mL/m^2^ (78.4% versus 92.2%, *P* = 0.003) in model 4. Kaplan-Meier survival curve showed this significant difference among groups according to the combined criteria (the E/e’ ratio quartile and LVSVI) (log-rank test, P = 0.027) (Fig 2D). The hazard ratios and 95% CIs of the combination of LVEF and E/A ratio and combination of SVI and E/e’ ratio are shown in Table 5.

## Discussion

There were two main findings of our current study. First, reduced LVEF and a decreased transmitral E/A ratio from the quantitative quartile analysis were found to be independent predictors of late overall mortality after LT, even after adjusting for clinically relevant variables. Second, the combination of LVSF and LVDF revealed a higher mortality rate than LV systolic dysfunction (LVSD) or LVDD alone.

Preoperative cardiac dysfunction is an independent risk factor for adverse cardiovascular events and mortality after cardiac and non-cardiac surgery [16–20]. Likewise, several studies have reported that pretransplant cardiac dysfunction is a major predictor of adverse outcomes after LT [7, 8, 10, 21]. A recent large national study that analyzed the recipients of primary LT in the Organ Procurement and Transplantation Network database also supported the importance of preoperative cardiac dysfunction by showing that cardiovascular disease death was the leading cause of early mortality (40%), followed by infection (28%) and graft failure (12%) [22]. However, few studies have quantitatively stratified the cardiac risk of LT candidates using pretransplant Doppler echocardiography to predict longer term all-cause mortality.

### LV systolic function

Of the parameters reflecting LV systolic function, non-survivors at 4 years in our present study series had a lower LVEF than survivors (median, 63% and 65%; *P* = 0.025) and LVEF was an independent predictor of overall 4-year mortality. However, unlike mortality at 4 years, the mortality rates at 3 months and 1 year were not significantly different between the LVEF>60% and LVEF≤60% groups (7.7% and 13.8%, respectively; *P* = 0.015). These results suggested that the prediction potential of LVEF is limited to late mortality following LT. Previous studies also supported these findings, echoing our conclusions that the systolic function of ESLD patients would not have a discrimination potential in early outcome prediction. For example, Dowsely et al.[7] reported that the pretransplant LVEF did not differ between an early heart failure group and a control group (median, 69% and 69%, *P* = 0.30) after LT. In addition, Ruiz-del-Arbol et al.[10] found that LVEF was significantly lower (mean, 70% and 75%; *P* = 0.050) in ESLD patients who died than in those who survived, but it was not a predictor of 1-year mortality in multivariable analysis, as in our study. Importantly, especially given that outcomes such as heart failure and death are clinically critical events, their LVEF results of 69% and 70% are unexpectedly high. Likewise, our LVEF of 63% at 4 years to predict mortality is also considered high.

In ESLD patients, it is well recognized that resting LVEF is a poor index of LVSF because impaired systolic function does not manifest until physiological, pharmacological, or surgical stressors are imposed [4, 11]. Classically, the threshold of an abnormal LVEF is 50% or 55% according to the guidelines of the ASE and European Society of Echocardiography [23, 24], whereas LVEF<55% is adopted in the 2005 WGO cirrhotic cardiomyopathy criteria. However, most patients with ESLD have a normal or supranormal LVEF because cirrhotic cardiomyopathy is characterized by a hyperdynamic circulatory state with high cardiac output at rest due to a low systemic vascular resistance [4, 25, 26]. Considering this pathophysiology and the results of present and previous studies, changing the lower limit of normality is a potential method to define LVSD in cirrhotic cardiomyopathy. However, a more detailed classification of the cardiac dysfunction of ESLD patients is required.

In this regard, the cardiac function of LT candidates with LVEF>55% according to WGO guidelines might be the subject of controversy, namely, whether cardiac systolic function above this value is still predictive of early and late outcomes. Indeed, in our current study, patients with an abnormal LVEF, according to the conventional criteria of LVEF≤55%, comprised 1.7% of our series while those with LVEF<55% comprised only 0.2%. In addition, all of our patients showed an LVEF>50%. This finding is consistent with those of other studies. In Raevens et al. [9], 2% of LT recipients (*n* = 137) were categorized as a systolic dysfunction group according to an LVEF<55% in the WGO echocardiographic guidelines. Dowsely et al.[7] and Ruiz-del-Arbol et al.[10] also found that all patients in their study cohorts (*n* = 107 and 80, respectively) had a normal LVEF (>50%). Hence, we stratified patients according to an LVEF≤60%, which was the lowest LVEF quartile in our study cohort, although it is higher than the standard criterion used by major societies. Nonetheless, we successfully showed that patients with an LVEF≤60% had higher late mortality than those with an LVEF>60%, even after adjusting for clinically relevant variables (hazard ratio = 1.90, 95% CI = 1.15–3.15, *P* = 0.012). Therefore, our results suggest that stratification of ESLD patients with an LVEF≤60% is recommended to predict late mortality after LT, in contrast to the ASE and WGO guidelines of LVEF<55%. Given that post-transplantation immunosuppressive drugs and other medications may contribute to the progression of cardiac and atherosclerotic vascular changes [27] it is plausible that an LVEF≤60% could be used to identify populations at higher risk of 4-year mortality after LT.

### LV Diastolic function

Increasing numbers of recent studies have emphasized the importance of LVDF in predicting poor early and late outcomes after LT [1, 7–9]. LVDD is the main feature of cardiac dysfunction in ESLD patients but its reported prevalence and outcomes vary widely because various definitions of LVDD have been used [1, 9, 10, 25]. Ruiz-del-Arbol et al.[10] reported that LVDD was found in 66% of ESLD patients in their relatively small sample-sized study (*n* = 80) using a "practical approach" to grade LVDD according to previous ASE guidelines [12]. In their study, 53% of ESLD patients had grade 1 LVDD and 47% had grade 2; no patients had severe (grade 3) LVDD. They found that 1-year survival before LT differed according to the degree of LVDD (without LVDD, 95%; grade 1 LVDD, 79%; grade 2 LVDD, 39%; *P*<0.001). Meanwhile, Mittal et al.^1^ reported that LVDD was found in 19% of LT candidates (*n* = 970)—mild in 48%, moderate in 30%, and severe in 22%—and found that pretransplant LVDD is significantly associated with increased risk of allograft rejection, graft failure, and mortality.

In contrast, another report [9] showed that although the prevalence of LVDD was 43% (*n* = 173), mortality was not strongly affected by its presence. Notably, to date, the prevalence and classification of LVDD is highly variable. Accordingly, different outcomes may also be inevitable. Furthermore, some articles argue that current LVDD grading systems cannot classify every patient into an LVDD grade, depending on the definition used, and leave a substantial portion of patients as unclassified [28, 29]. Such results are likely because LVDD grading systems are complex to use and are considered to be somewhat imperfect with conventional echocardiographic variables alone [28, 30].

Collectively, inconsistencies in outcome studies after LT in ESLD patients are not so surprising given that (1) studies use incomplete grading systems, and (2) there may be considerable differences in the normal values of echocardiographic parameters between ESLD patients and the general population. Hence, we believe, as shown in our current analyses, that our criteria derived from quantitative quartile analysis could be useful for predicting all-cause mortality after LT.

Because TDI is an evolving echocardiographic tool and one of the most load-independent measurements of cardiac function, it plays an important role in prognosis assessment [11, 12, 14]. Recent studies have shown that TDI-derived parameters, such as the s’ velocity, e’ velocity, and E/e’ ratio, are useful prognosticators in various cardiovascular diseases and for numerous cardiac and non-cardiac operations [11]. In particular, patients with a TDI s’ velocity and e’ velocity of <3cm/s are predicted to have a poor prognosis and high risk of mortality or a cardiovascular event [11]. However, in our present study, three patients (0.4%) and one patient (0.1%) only had an s’ velocity and e’ velocity of <3cm/s, respectively. Furthermore, even in the 5th percentiles of TDI results, an s’ velocity of <6cm/s and e’ velocity of <5cm/s did not predict mortality. Therefore, our results again suggest that the echocardiographic characteristics of ESLD patients are quite different from those of non-ESLD patients.

Of the echocardiographic parameters indicating LVDD, an E/A ratio of <1, is a robust predictor of death in ESLD patients after transjugular intrahepatic portosystemic stent shunt insertion [31]. In our current study, an E/A ratio also predicted higher mortality in a dose-dependent manner after LT of up to 4 years. However, use of the E/A ratio alone to diagnose LVDD has been criticized because of its dependence on loading conditions and the effect of age [32]. Nonetheless, our current analysis of ESLD patients aged 18–60 years showed that a reduced E/A ratio is an independent predictor of mortality after LT. In addition, in our quartile analysis, patients with an E/A ratio<0.9 (Q1) had a 2.19-fold higher risk of death than those with an E/A ratio>1.4 (Q4), reemphasizing the importance of the E/A ratio as a predictor of mortality in the ESLD population.

### Combined use of LVSF and LVDF

Given that LVSF and LVDF are tightly interconnected, close monitoring of both cardiac function parameters is always recommended [33]. Because systolic heart failure is rarely seen in LT candidates and LVSD is masked by the peripheral vasodilation, it is expected that LVSD parameters alone would be a weak predictor of mortality in ESLD patients. Hence, we hypothesized that LT candidates with combined LVDD and LVSD would have a worse prognosis after LT. Indeed, we firstly showed that patients with both LVSF and LVDF had a higher mortality rate than those with LVSD or LVDD alone. In this regard, although the systolic parameter of the LVSVI and diastolic parameter of the E/e’ ratio were not significant predictors of mortality after LT, we found that combined use of systolic and diastolic function (the LVSVI plus E/e’ ratio) could predict a poorer prognosis after LT, even after adjusting for the clinical variables of the CTP score and total bilirubin and creatinine levels. Specifically, we used an E/e’ ratio>11.5 from quartile analysis as a cut-off value which is lower than usual criteria used in the general population. It is sufficiently convincing, given that an E/e’ ratio>10 is able to predict pretransplant mortality at 1 year and development of heart failure early after LT [7, 10]. Additionally, our results also revealed that combined use of LVEF and E/A ratio is a strong predictor of mortality. Thus, the combined criteria used in our current study might serve as a novel tool to assess prognosis in LT candidates.

### Limitations

This was a single-center retrospective study and, therefore, it is possible that the management strategy before LT at our institution may have affected the cardiac systolic and diastolic function and that the intraoperative and postoperative management may have affected the survival rate. In addition, although the echocardiographic image of our institution known to be reliable and adhere to the American Society of Echocardiography guideline [23, 34], we did not assess reproducibility test owing to its retrospective nature. Second, we included only patients aged 18–60 years due to the high prevalence of LVDD in elderly patients and excluded patients with comorbidities and patients who did not undergo preoperative Doppler echocardiography, which may have caused a selection bias for LT candidates with LVSF and LVDF. However, our study enrolled a sufficiently sized cohort and we adjusted for patient age in each statistical model. Third, we used E/A ratio for classifying patients’ diastolic function, although the E/A ratio is a ‘two face’ parameter which is representative as a pseudonormal pattern. However, given that the majority of the patients were classified as normal (80.1%) or indeterminate (15.7%) diastolic function in our study according to the recent guideline [35], the high E/A ratio mostly indicates that normal diastolic function rather than high E/A pattern which is seen in severe diastolic dysfunction. Fourth, several sonographers and cardiologists measured the echocardiographic data. Thus, we cannot exclude some interobserver variability with regard to these results.

## Conclusions

The combination of LVSF and LVDF is a better predictor of survival than LVSF or LVDF alone. This approach might help to stratify the cardiac risk of LT candidates using pretransplant Doppler echocardiography and better predict all-cause mortality. A careful follow-up of LT recipients is crucial because pretransplant quantification of cardiac dysfunction can help to predict poor survival.

## Supporting information

S1 Table Clinical and Echocardiographic characteristics according to the E/A ratio and LV ejection fraction. (DOCX)

S1 File Study data set. (CSV)

## Abbreviations

A: late transmitral flow velocity
ASE: American Society of Echocardiography
CTP: Child-Turcotte-Pugh
DT: deceleration time
E: early transmitral flow velocity
EDV: end-diastolic volume
EF: ejection fraction
ESLD: end-stage liver disease
ESV: end-systolic volume
LT: liver transplantation
LA: left atrium
LVDD: left ventricular diastolic dysfunction
LVDF: left ventricular diastolic function
LVSD: left ventricular systolic dysfunction
LVSF: left ventricular systolic function
MELD: Model for End-stage Liver Disease
SV: stroke volume
SVI: stroke volume index
TDI: tissue Doppler imaging
WGO: World Gastroenterology Organization
