# Correction: Prediction of all-cause mortality after liver transplantation using left ventricular systolic and diastolic function assessment

**DOI:** 10.1371/journal.pone.0213967

**Published:** 2019-03-13

**Authors:** The PLOS ONE Staff

The affiliation for the second, third and sixth authors is incorrect. Jung-Won Kim, Yun-Sic Bang and Bo-Hyun Sang are not affiliated with #2 but with #3: Department of Anesthesiology and Pain Medicine, CHA Bundang Medical Center, CHA University, Seongnam, Republic of Korea. The publisher apologizes for this error.
