# Supplementary material for: Prediction of all-cause mortality after liver transplantation using left ventricular systolic and diastolic function assessment
Source: PLoS One. 2019 Jan 25;14(1):e0209100. doi: 10.1371/journal.pone.0209100 (PMC6347358; doi:10.1371/journal.pone.0209100)
Supplement: S1 Table — (DOCX) [file pone.0209100.s001.docx]

**S1 Table. Clinical and Echocardiographic Characteristics According to the E/A ratio and LV Ejection Fraction**

|  | | **E/A ratio** | | | | ***P*** | **Ejection fraction** | | ***P*** |
| --- | --- | --- | --- | --- | --- | --- | --- | --- | --- |
| **Variables** | | **Quartile 1**  **(<0.9)** | **Quartile 2**  **(0.9-1.1)** | **Quartile 3**  **(1.2-1.4)** | **Quartile 4**  **(>1.4)** | **for trend** | **>60%** | **≤60%** | **for trend** |
| Age (y) | | 53 (49-57) | 52 (47-55) | 51 (45-55) | 49 (42-53) | <0.001 | 51 (46-55) | 52 (47-56) | 0.575 |
| Male | | 174 (80.6%) | 152 (74.1%) | 153 (73.6%) | 165 (78.6%) | 0.599 | 509 (75.0%) | 135 (84.4%) | 0.011 |
| Body mass index (kg/m^2^) | | 23.5 (21.6-26.3) | 23.5 (21.3-26.0) | 24.7 (22.0-26.8) | 23.7 (21.4-26.2) | 0.673 | 23.6 (21.7-26.1) | 24.0 (21.3-26.6) | 0.602 |
| Hypertension | | 39 (18.1%) | 25 (12.2%) | 23 (11.1%) | 10 (4.8%) | <0.001 | 74 (10.9%) | 23 (14.4%) | 0.216 |
| Diabetes mellitus  Ischemic heart disease | | 57 (26.4%)  32 (14.8%) | 48 (23.4%)  25 (12.2%) | 39 (18.8%)  30 (14.4%) | 33 (15.7%)  25 (11.9%) | 0.003  0.528 | 140 (20.6%)  87 (12.8%) | 37 (23.1%)  25 (15.6%) | 0.485  0.347 |
| Varix bleeding | | 49 (22.7%) | 50 (24.4%) | 47 (22.6%) | 53 (25.2%) | 0.651 | 160 (23.6%) | 39 (24.4%) | 0.828 |
| MELD score | | 15 (10-27) | 14 (10-22) | 14 (9-21) | 14 (10-23) | 0.324 | 14 (10-23) | 14 (9-20) | 0.305 |
| Child-Turcotte-Pugh score | | 9 (6-11) | 8 (6-11) | 8 (6-10) | 8 (6-10) | 0.275 | 9 (6-11) | 8 (6-10) | 0.034 |
| Child-Turcotte-Pugh class | |  |  |  |  | 0.279 |  |  | 0.099 |
|  | A | 60 (27.8%) | 58 (28.3%) | 62 (29.8%) | 57 (27.1%) |  | 185 (27.2%) | 52 (32.5%) |  |
|  | B | 65 (30.1%) | 69 (33.7%) | 77 (37.0%) | 80 (38.1%) |  | 234 (34.5%) | 57 (35.6%) |  |
|  | C | 91 (42.1%) | 78 (38.0%) | 69 (33.2%) | 73 (34.8%) |  | 260 (38.3%) | 51 (31.9%) |  |
| Medication | |  |  |  |  |  |  |  |  |
|  | Diuretics | 66 (30.6%) | 58 (28.3%) | 57 (27.4%) | 53 (25.2%) | 0.221 | 199 (29.3%) | 35 (21.9%) | 0.059 |
|  | Beta blockers | 36 (16.7%) | 29 (14.1%) | 42 (20.2%) | 37 (17.6%) | 0.450 | 122 (18.0%) | 22 (13.8%) | 0.203 |
| Donor type | |  |  |  |  | 0.241 |  |  | 0.542 |
|  | Cadaveric | 38 (17.6%) | 23 (11.2%) | 22 (10.6%) | 29 (13.8%) |  | 93 (13.7%) | 19 (11.9%) |  |
|  | Living | 178 (82.4%) | 182 (88.8%) | 186 (89.4%) | 181 (86.2%) |  | 586 (86.3%) | 141 (88.1%) |  |
|  | Graft-recipient weight ratio | 1.03 (0.89-1.20) | 1.00 (0.84-1.18) | 0.97 (0.86-1.14) | 0.96 (0.84-1.13) | 0.052 | 0.99 (0.85-1.17) | 0.97 (0.86-1.13) | 0.876 |
| Hemodynamic data | |  |  |  |  |  |  |  |  |
|  | Systolic blood pressure (mmHg) | 114 (101-125) | 110 (99-121) | 108 (101-119) | 108 (99-120) | 0.021 | 110 (99-120) | 112 (102-122) | 0.164 |
|  | Diastolic blood pressure (mmHg) | 72 (64-81) | 69 (63-77) | 71 (62-77) | 67 (61-74) | <0.001 | 69 (62-77) | 71 (64-80) | 0.031 |
|  | QTc (ms) | 452 (432-477) | 445 (426-468) | 448 (429-469) | 449 (429-472) | 0.448 | 449 (430-470) | 448 (424-471) | 0.544 |
| Laboratory data | |  |  |  |  |  |  |  |  |
|  | Hemoglobin (g/dL) | 10.6 (9.1-12.8) | 10.5 (9.2-12.2) | 11.0 (9.2-12.8) | 10.4 (9.0-12.2) | 0.259 | 10.4 (9.0-12.2) | 11.1 (9.9-12.9) | 0.005 |
|  | Platelet (×10^3^/µl) | 63 (42-93) | 56 (36-83) | 58 (39-85) | 55 (38-76) | 0.019 | 57 (38-83) | 61 (41-95) | 0.109 |
|  | Total bilirubin (mg/dL) | 3.2 (1.3-16.2) | 2.2 (1.2-7.8) | 2.6 (1.2-6.9) | 2.8 (1.4-9.9) | 0.179 | 2.8 (1.4-11.5) | 2.5 (1.2-6.3) | 0.061 |
|  | Albumin (g/dL) | 3.2 (2.8-3.6) | 3.1 (2.6-3.6) | 3.1 (2.7-3.6) | 3.1 (2.7-3.5) | 0.434 | 3.1 (2.7-3.6) | 3.1 (2.6-3.7) | 0.828 |
|  | Creatinine (mg/dL) | 0.9 (0.7-1.1) | 0.8 (0.6-1.0) | 0.7 (0.6-0.9) | 0.7 (0.6-0.9) | 0.002 | 0.8 (0.6-1.0) | 0.8 (0.6-1.0) | 0.381 |
|  | Prothrombin time (INR) | 1.47 (1.21-1.91) | 1.45 (1.19-1.86) | 1.43 (1.20-1.84) | 1.43 (1.24-1.88) | 0.600 | 1.47 (1.22-1.94) | 1.42 (1.19-1.72) | 0.229 |
|  | BNP (pg/mL) | 46 (17-112) | 38 (18-91) | 48 (21-106) | 53 (23-139) | 0.170 | 51 (20-117) | 29 (16-85) | 0.180 |
|  | Log BNP (pg/mL) | 3.9 ± 1.4 | 3.8 ± 1.3 | 3.8 ± 1.3 | 4.0 ± 1.4 | 0.170 | 4.0 ± 1.3 | 3.6 ± 1.3 | 0.003 |
| Echocardiographic data | |  |  |  |  |  |  |  |  |
|  | LVEDV index (mL/m^2^) | 54.7 (47.6-63.6) | 61.6 (52.9-71.1) | 63.5 (55.6-75.0) | 67.3 (58.1-77.7) | <0.001 | 62.0 (52.9-72.6) | 64.1 (53.5-72.4) | 0.600 |
|  | LVESV index (mL/m^2^) | 19.4 (16.2-24.2) | 21.7 (18.9-26.0) | 22.9 (19.4-27.7) | 23.6 (19.9-28.3) | <0.001 | 21.4 (17.9-25.1) | 26.0 (22.0-30.7) | <0.001 |
|  | LVMI (g/m^2^) | 92.0 (80.4-103.5) | 86.3 (76.3-99.1) | 90.5 (79.7-102.9) | 93.7 (80.7-107.6) | 0.011 | 90.1 (78.5-102.8) | 93.8 (81.0-111.6) | 0.022 |
|  | PGsys(RV-RA) (mmHg) (n=711) | 21.0 (18.0-25.0) | 21.0 (18.0-25.0) | 23.0 (19.0-27.0) | 23.0 (19.0-27.0) | <0.001 | 21.0 (19.0-27.0) | 21.0 (18.0-25.0) | 0.246 |
| Systolic function | |  |  |  |  |  |  |  |  |
|  | Fractional shortening (%) | 41.7 ± 6.5 | 41.3 ± 5.7 | 40.8 ± 5.5 | 39.8 ± 5.9 | 0.089 | 41.3 ± 5.9 | 39.1 ± 5.6 | <0.001 |
|  | LVSV index (mL/m^2^) | 36.0 (30.3-41.3) | 39.7 (33.1-44.8) | 41.0 (35.1-49.0) | 42.9 (37.7-50.1) | <0.001 | 41.1 (35.1-48.0) | 37.7 (31.4-42.1) | <0.001 |
|  | LVEF (%) | 64.0 (61.0-68.0) | 64.0 (61.5-67.0) | 64.0 (61.0-67.0) | 65.0 (62.0-67.0) | 0.236 | 65.0 (63.0-68.0) | 59.0 (58.0-60.0) | <0.001 |
|  | s’ (cm/s) | 8.8 (7.6-10.0) | 8.4 (7.5-9.8) | 8.5 (7.3-9.6) | 8.2 (7.3-9.1) | 0.002 | 8.4 (7.6-9.7) | 7.9 (7.0-9.0) | <0.001 |
| Diastolic function | |  |  |  |  |  |  |  |  |
|  | LA diameter index (mm/m^2^) | 22.3 (20.3-24.6) | 22.7 (21.0-24.7) | 22.6 (20.9-24.7) | 23.1 (21.2-25.5) | 0.032 | 22.8 (20.9-24.9) | 22.2 (20.1-24.9) | 0.043 |
|  | E/A ratio | 0.82 (0.74-0.87) | 1.05 (0.98-1.11) | 1.28 (1.22-1.35) | 1.70 (1.56-1.90) | <0.001 | 1.20 (0.94-1.50) | 1.18 (0.93-0.42) | 0.591 |
|  | DT (ms) | 214 (180-247) | 210 (184-236) | 200 (179-225) | 194 (170-214) | <0.001 | 205 (179-231) | 195 (173-222) | 0.080 |
|  | e’ (cm/s) | 6.5 (5.6-7.5) | 7.3 (6.3-8.5) | 8.1 (6.9-9.1) | 9.0 (7.9-10.1) | <0.001 | 7.9 (6.6-9.1) | 7.8 (6.5-9.0) | 0.166 |
|  | a’ (cm/s) | 10.0 (8.7-11.8) | 9.5 (8.5-11.0) | 9.0 (7.8-10.3) | 7.9 (7.0-8.9) | <0.001 | 9.0 (7.9-10.8) | 8.5 (7.6-9.6) | 0.001 |
|  | e’/a’ ratio | 0.62 (0.53-0.75) | 0.76 (0.64-0.90) | 0.87 (0.73-1.09) | 1.16 (0.95-1.36) | <0.001 | 0.82 (0.67-1.10) | 0.89 (0.69-1.11) | 0.297 |
|  | E/e’ ratio | 9.0 (7.5-11.0) | 9.6 (8.1-11.6) | 9.5 (8.0-11.9) | 10.1 (8.3-11.9) | 0.002 | 9.7 (8.1-11.7) | 9.1 (7.7-11.6) | 0.094 |
| Outcome | |  |  |  |  |  |  |  |  |
|  | 3-month mortality | 11 (5.1%) | 6 (2.9%) | 3 (1.4%) | 4 (1.9%) | 0.03 | 19 (2.8%) | 5 (3.1%) | 0.824 |
|  | 1-year mortality | 21 (9.7%) | 17 (8.3%) | 7 (3.4%) | 9 (4.3%) | 0.005 | 40 (5.9%) | 14 (8.8%) | 0.185 |
|  | 4-year mortality | 27 (12.5%) | 22 (10.7%) | 13 (6.3%) | 12 (5.7%) | 0.004 | 52 (7.7%) | 22 (13.8%) | 0.015 |

Data are expressed as median (interquartile range), mean ± standard deviation, or number (percentage).

Abbreviations: MELD, Model for End-stage Liver Disease; QTc, corrected QT interval; INR, international normalized ratio; BNP, brain natriuretic peptide; LVMI, left ventricular mass index; PGsys (RV-RA), systolic pressure gradient between right ventricle and right atrium; LVEDV, left ventricular end-diastolic volume; LVESV, left ventricular end-systolic volume; LVSV, left ventricular stroke volume; LVEF, left ventricular ejection fraction; s’, systolic myocardial velocity; LA, left atrium; E, early transmitral flow velocity; A, late transmitral flow velocity; DT, deceleration time of E; e’, early diastolic myocardial velocity; a’, late diastolic myocardial velocity.
